# Traitement de la fracture vertébrale ostéoporotique par kyphoplastie percutanée avec un extenseur de type SpineJack®

**DOI:** 10.11604/pamj.2020.35.136.21296

**Published:** 2020-04-22

**Authors:** Moussa Diallo, Romuald Kouitcheu, Adamou Touta, Jean-Marc Kaya, Lucas Troude, Anthony Mélot, Pierre-Hugues Roche

**Affiliations:** 1 Service de Neurochirurgie, Hôpital Nord Marseille, Marseille, France; 2 Service de Neurochirurgie, CHU Gabriel Touré, Faculté de Médecine Université de Bamako (USTTB), Bamako, Mali; 3 Service de Neurochirurgie CHU Yopougon, Faculté de Médecine Université FHB Abidjan, Abidjan, Cote d'Ivoire

**Keywords:** Fracture vertébrale, kyphoplastie, ostéoporose, SpineJack®, Vertebral fracture, kyphoplasty, osteoporosis, SpineJack®

## Abstract

**Introduction:**

L'objectif était d’évaluer le résultat de notre prise en charge chirurgicale des fractures ostéoporotiques vertébrales avec un extenseur vertébral percutané de type SpineJack®.

**Méthodes:**

Il s'agit d'une étude rétrospective analytique et mono centrique de 33 mois (avril 2015 - décembre 2017). Elle avait porté sur les patients ayant été traités par kyphoplastie avec le pour une fracture vertébrale ostéoporotique. Le kit comprenant un extenseur vertébral en titane à type de SpineJack® de Vexim et le ciment acrylique avait été utilisé. Les patients présentant une rachialgie d'intensité croissante malgré le traitement lié à une fracture vertébrale ostéoporotique ont été inclus dans l'étude ainsi que ceux ayant acceptés le principe de la chirurgie et donner leur consentement éclairé. La fracture vertébrale était diagnostiquée à la tomodensitométrie (TDM) et son caractère récent confirmé par l'imagerie par résonnance magnétique (IRM).

**Résultats:**

Entre avril 2015 et décembre 2017, trente-sept patients porteurs de fractures ostéoporotiques vertébrales ont été traités par kyphoplastie avec un extenseur vertébral en titane. L'âge moyen était de 73,4 ans avec un sexe ratio à 0,6. L'échelle visuelle analogique moyenne était de 7,3. Le score d'Osvestry était en moyenne de 81,6. Les fractures étaient prédominantes au niveau de la charnière thoraco-lombaire. L'angle de cyphose vertébrale mesurait en moyenne 18,45°. La kyphoplastie avait concerné 44 vertèbres. Quatre cas de complications opératoires avaient été enregistrés. La durée moyenne d'hospitalisation était de 5,4 jours. A 6 mois de suivi, 9 patients étaient encore sous traitement antalgique. A un an, aucun cas de fracture de vertèbre adjacente n'avait été trouvé.

**Conclusion:**

La kyphoplastie percutanée avec un extenseur en titane est un moyen thérapeutique sûr et efficace des fractures vertébrales ostéoporotiques. Avec son effet quasi immédiat, il permet au patient, un retour rapide à la vie active.

## Abstract (en)

**Abstract:**

The purpose of this study was to assess the outcome of surgical management of osteoporotic vertebral fractures with percutaneous kyphoplasty using expandible SpineJack® implant.

**Methods:**

We conducted an analytical retrospective single-center study over a period of 33-months (April 2015 - December 2017). It involved patients treated with kyphoplasty for an osteoporotic vertebral fracture. The kit consisted of a Vexim SpineJack® expandable titanium implant and acrylic cement. Patients with back pain increasing in intensity despite treatment for an osteoporotic vertebral fracture were included in the study as well as those who had undergone surgery and had given their informed consent. The vertebral fracture was diagnosed based on CT scan results and its recent state was confirmed by MRI.

**Results:**

Between april 2015 and december 2017, thirty-seven patients with osteoporotic vertebral fractures were treated with kyphoplasty using expandable titanium vertebral implant. The average age of patients was 73.4 years, with a sex ratio of 0.6. Mean visual analog scale score was 7.3. Mean Oswestry score was 81.6. The patients mainly had fractures of the thoracolumbar hinge. Mean vertebral kyphotic angle was 18.45°. Kyphoplasty was used for the treatment of 44 vertebrae. Four patients had surgical complications. The average length of stay in hospital was 5.4 days. After a follow-up period of 6 months, 9 patients were still receiving analgesic therapy. After one year, no adjacent level fracture was found.

**Conclusion:**

Percutaneous kyphoplasty using expandible titanium implant is a safe and effective therapeutic mean for osteoporotic vertebral fractures. Given its almost immediate result, patients can rapidly get back to their active lives.

## Introduction

L'accroissement de l'espérance de vie a permis de constater une augmentation de la fréquence des fractures ostéoporotiques vertébrales dans la population générale [1-3] avec des conséquences non négligeables. La gravité est liée au caractère invalidant des douleurs rachidiennes et au trouble de l'équilibre sagittal inhérent à la cyphose vertébrale croissante [4]. La kyphoplastie est une technique chirurgicale mini-invasive utilisée pour le traitement des fractures vertébrales en compression. Par extension, elle a été appliquée aux fractures ostéoporotiques sur la base des données issues de la vertébroplastie. Le SpineJack est l'un des outils de troisième génération utilisés dans la prise en charge des fractures tassement vertébrales. Le but de cette étude était évaluer le résultat de notre prise en charge chirurgicale des fractures ostéoporotiques vertébrales avec un extenseur vertébral percutané de type SpineJack®.

## Méthodes

Il s'agit d'une étude rétrospective analytique et mono centrique de 33 mois (avril 2015-décembre 2017) réalisée dans le Service de Neurochirurgie de l'Hôpital Nord à Marseille. Elle avait porté sur les patients traités par kyphoplastie avec un extenseur vertébral de type SpineJack® pour une fracture vertébrale d'origine ostéoporotique. Les critères d'inclusion à cette étude étaient les suivants: la présence de rachialgie d'intensité croissante liée à une fracture vertébrale ostéoporotique malgré le traitement antalgique; la fracture vertébrale diagnostiquée à la tomodensitométrie (TDM) et le caractère récent confirmé par l'imagerie par résonnance magnétique (IRM); le diagnostic de l'ostéoporose fait par l'ostéodensitométrie; l'acceptés le principe de la chirurgie par le patient et consentement éclairé obtenu. Ont été exclus de cette étude, les autres indications de la kyphoplastie percutanée en dehors de la fracture ostéoporotique; la kyphoplastie percutanée par avec d'autres dispositifs que le SpineJack; la kyphoplastie à foyer ouvert; la kyphoplastie percutanée associée à une arthrodèse rachidienne; les patients n'ayant pas adhéré aux principes du traitement; les patients ayant opté pour le traitement conservateur. L'âge, le sexe, l'échelle visuelle analogique (EVA), la durée d'évolution des symptômes, le score d'Osvestry (ODI) évalué sur 100 points, le siège de la fracture, l'angle de cyphose vertébrale, le nombre vertèbres traitées lors d'une intervention, la durée la chirurgie, les complications opératoires, le suivi et le devenir des patients étaient les variables étudiées. Le test de khi-carré a été utilisé pour comparer les variables quantitatives. Le résultat du test était significatif pour une valeur de p < 0,05.

**Protocole chirurgical:** le patient sous anesthésie générale était installé en décubitus ventral sur deux billots (un iliaque et un pectoral). Un repérage de face et de profil de la vertèbre à traiter de ses deux pédicules était réalisé sous amplificateur de brillance. Après asepsie et la pose des champs avec l'amplificateur en place, une paire de trocart de Jamshidi était introduite dans la vertèbre par voie trans-pédiculaire à travers une incision cutanée de 3 millimètres (mm) sous contrôle radioscopique. Le retrait du trocart était fait sur une broche-guide en place. Celle-ci servira de tuteur à la chemise de travail et au taraud qui fera la voie de passage à l'implant (le SpineJack®). L'alésage permet de faire la chambre d'implantation du dispositif. Une paire de SpineJack® introduite dans le corps vertébral permettait le redressement de la hauteur de celui-ci par soulèvement douce des plateaux vertébraux par l'ouverture du SpineJack (extension) et le déploiement de ses ailettes. L'injection du ciment acrylique à base de polyméthylméthacrylate acide (PMMA) à l'intérieur de la vertèbre se faisait autour du dispositif sous contrôle radioscopique. La polymérisation de ce ciment renforcera le dispositif et consolidera la vertèbre fracturée. Un point de suture au niveau de chaque incision terminait la chirurgie.

**Les suites opératoires:** les patients sont levés le lendemain de la chirurgie, un scanner de contrôle est effectué avant leur sortie.

## Résultats

**Figure 1:**
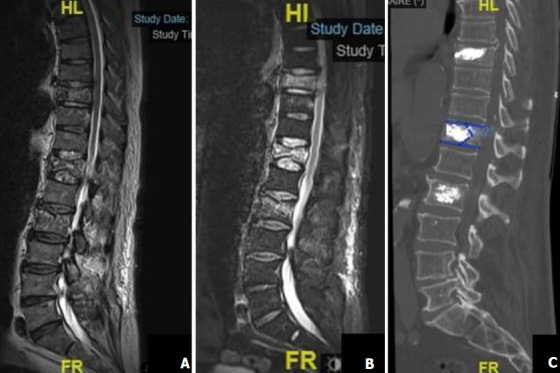
Imagerie du rachis en coupe sagittale IRM en séquence T2 sagittale, fracture tassement des vertèbres T12, T9 et L2 (A) IRM en séquence T2 STIR, aspect hypersignal des vertèbres T12, T9 et L2 (B) TDM de contrôle après kyphoplastie (C)

**Figure 2:**
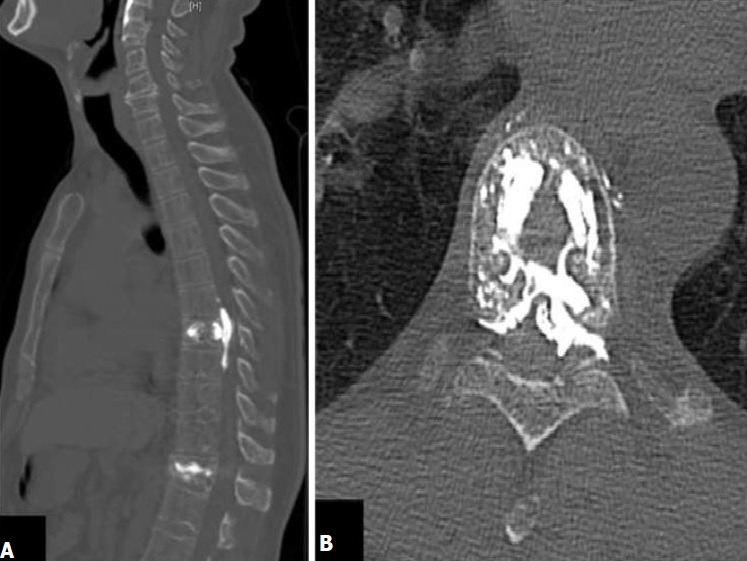
TDM de contrôle postopératoire coupe sagittale, fuite du ciment dans l'espace épidural thoracique (A), coupe axiale, fuite épidurale et foraminale gauche du ciment acrylique (B)

**Figure 3:**
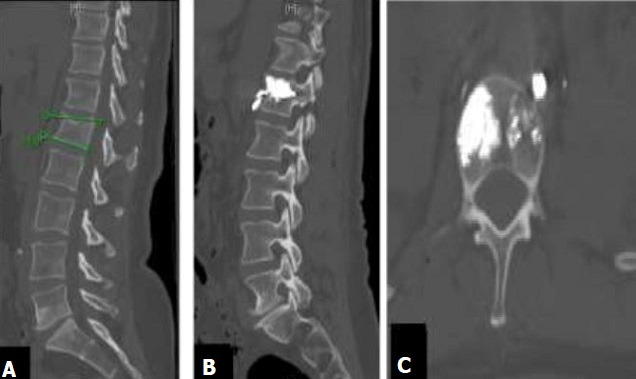
TDM pré et postopératoire du rachis coupe sagittale, fracture tassement vertèbre L1 (A), coupe sagittale, fuite pré-vertébrale du ciment après kyphoplastie (B), coupe axiale, fuite du ciment en pré vertébrale gauche (C)

Entre avril 2015 et décembre 2017, deux cent soixante neufs patients porteurs de fracture rachidienne ont été traités par kyphoplastie percutanée avec le SpineJack® parmi lesquels 37 cas de fractures ostéoporotiques. La moyenne d'âge de ces patients était de 73,4 ans avec des extrêmes de 52 ans et 98 ans. Le sexe féminin était prédominant (59,5%) avec un sexe ratio de 0,6. La douleur était le maître-symptôme. L'échelle visuelle analogique (EVA) préopératoire était en moyenne de 7,3 (extrêmes 6 et 10). La durée moyenne d'évolution de ces douleurs rachidiennes était de 14 ±2,5 jours (extrêmes 6 et 29 jours). Chez 35,1% de nos patients (13 cas), la douleur avait un caractère invalidant mais sans trouble neurologique associé. Le score d'Osvestry (ODI) était en moyenne de 81,6 (extrêmes 55 et 97). Avant le traitement chirurgical, 62,6% des patients étaient sous un traitement co-antalgique et 16% avaient un traitement antalgique de palier 3. Les détails du traitement antalgique des patients sont consignés dans le Tableau 1. La radiographie standard du rachis de face et de profil avait été réalisée chez 29 patients (78,4%). Tous nos patients avaient passé une TDM du rachis thoracique et lombaire sans injection pour le diagnostic de la fracture vertébrale. Une IRM complémentaire préopératoire (Figure 1 A,B) était réalisée pour confirmer le caractère récent de la fracture. Ces examens avaient permis de trouver 44 fractures vertébrales dont 18 en région thoraciques et 26 en lombaires. L'atteinte prédominait au niveau de la charnière thoraco-lombaire T11à L2 avec une fréquence de 59%. La région lombaire était la plus touchée par rapport au siège thoracique (Tableau 2). La cyphose vertébrale mesurait en moyenne 18,45° (extrême 8,4 et 39,9°) sur l'imagerie diagnostique. La kyphoplastie avait concerné 44 vertèbres. Dans le même temps opératoire, le traitement avait concerné une seule vertèbre chez 25 patients 67,6% des cas; deux vertèbres chez 9 patients 24,3% et trois vertèbres chez 3 patients 8,1% (Figure 1 C). La durée moyenne de la chirurgie était de 19,9 min (extrêmes 13 min et 33 min). Quatre (04) cas de complications opératoires 8,1% avaient été enregistrés. Il s'agissait d'une fuite du ciment acrylique chez 3 patients et d'un cas d'hématome sous cutané. Les fuites du ciment concernaient l'espace épidural 1 cas (Figure 2), la région pré vertébrale 1 cas (Figure 3) et espace discal 1 cas.

**Tableau 1:** Répartition selon le traitement antalgique pré et postopératoire

Traitement antalgique	Préopératoire		Postopératoire	
	Effectif	Fréquence (%)	Effectif	Fréquence (%)
Pas d’antalgique	0	0	8	21,6
Palier 1	-	0	11	29,8
Palier 2	8	21,6	8	21,6
Co-antalgique	23	62,6	7	18,9
Palier 3	6	16,2	3	8,1
**Total**	**37**	**100**	**37**	**100**

**Tableau 2:** Répartition des patients selon le siège de la fracture vertébrale

Siège fracture		T8	T9	T10	T11	T12	total
Thoracique	Effectif	2	3	2	4	7	**18**
	Fréquence (%)	4,6	6,8	4,6	9,1	15,9	**41**
**Siège fracture**		**L1**	**L2**	**L3**	**L4**	**L5**	**total**
Lombaire	Effectif	8	7	4	4	3	**26**
	Fréquence (%)	18,1	15,9	9,1	9,1	6,8	**59**

Tous les patients avaient repris l'activité de marche le lendemain de la chirurgie. L'EVA postopératoire était en moyenne de 4,4 (extrêmes 2 et 3). La différentielle d'avec celle d'avant la chirurgie était de 2,9 avec un p = 0,008. L'ODI était de 21,2 (extrêmes 13 et 29) avec une différentielle de 60,4 d'avec celui du préopératoire p=0,03. L'angle de cyphose postopératoire était de 6,08° (extrêmes 2 et 13,7°). En moyenne, la chirurgie avait permis d'obtenir une correction moyenne de 12,4° de cyphose. La durée moyenne d'hospitalisation était de 5,4 jours (extrêmes 1 et 20 jours). Chez 21 patients 77,8%, cette hospitalisation n'avait pas excédé 3 jours. Vingt-quatre patients 64,9% étaient sortis à domicile ou en maison de retraite; 24,3% en centre de convalescence (9 cas) et 10,8% transférés dans un autre service (4 cas). Le délai moyen de suivi était de 8,08 mois (2 et 24 mois). A 6 mois de suivi, 5 patients étaient perdus de vue. Neuf patients étaient toujours sous traitement antalgique. Ils étaient repartis en 5 cas (15,6%) sous antalgiques de palier 1; deux patients (6,2%) sous palier 2 et deux co-antalgiques paliers. A un an d'évolution, 3 patients étaient sous antalgique de palier 2. Aucun cas de fractures de vertèbres adjacentes n'avait été trouvé. Les patients ayant eu une complication opératoire n'avaient présenté de trouble neurologique postopératoire immédiat ou tardif. L'efficacité thérapeutique était de 71,9% dans notre cohorte avec un taux de mortalité nul.

## Discussion

L'ostéoporose constitue un problème de santé publique eu égard à ses corolaires d'invalidités en cas de fractures vertébrales associées. Les fractures par compression vertébrale (FCV) sont des fractures ostéoporotiques les plus courantes dans le monde. Elles touchent 30 à 50% des personnes de plus de 50 ans. On estime à 1,4 million le nombre de patients FCV dans le monde et les taux d'incidence augmentent de façon exponentielle avec l'âge, en particulier chez les femmes [1, 2]. En Italie, la prévalence des fractures ostéoporotiques vertébrales en 2008 était estimée à environ 61 000, avec une augmentation de 6,3% sur 7 ans [3]. Les FCV entraînent fréquemment une morbidité importante et une utilisation importante des ressources de soins de santé [4]. Elles sont associées à une augmentation du risque de mortalité [5]. La TDM est incontournable au diagnostic de fracture vertébrale. Elle précise le nombre et le siège de la lésion. L'IRM permet de confirmer le caractère récent de la fracture par la présence d'une inflammation au sein du corps vertébral fracturé se traduisant par un hypersignal en séquence STIR. L'ostéodensitométrie rattache la fracture vertébrale à une étiologie ostéoporotique. Le traitement conservateur ayant montré ses limites, et l'arthrodèse par des vis et les tiges constitue une approche invasive avec un gros de débricollage du matériel lié à la fragilité des os, la chirurgie mini invasive est une solution adaptée. La kyphoplastie percutanée par un SpineJack est une technique mini-invasive utilisant les dispositifs de 3^e^ génération pour une reexpansion et une consolidation durable de la fracture vertébrale par compression. Les extenseurs vertébraux en titane permettent de prévenir la perte secondaire de hauteur de corps vertébral observée lors de la kyphoplastie avec ballonnet [6]. Au plan biomécanique, l'extension vertébrale par un extenseur en titane est supérieure à celle qui utilise le ballonnet en termes de restauration de l'équilibre sagittale et le maintien de la hauteur vertébrale [6-9]. Le SpineJack®, grâce à son mécanisme comparable à un cric permet de relever la vertèbre de l'intérieur via un acte de chirurgie mini-invasive. Il offre une solution efficace et rapide pour traiter ces fractures, tout en permettant de restaurer l'équilibre de la colonne vertébrale [7]. Cette restauration vertébrale optimale est obtenue par la grande ouverture du dispositif pouvant atteindre une hauteur maximale d'expansion de 17 mm. A notre avis, la restauration de la hauteur vertébrale et la consolidation de celle-ci par le ciment acrylique ont forcément un impact positif sur la douleur et l'équilibre sagittale de la colonne vertébrale. La chaleur dégagée par la polymérisation du ciment acrylique agirait sur les terminaisons des microfibres nerveuses au sein du corps vertébral. Dans leur cohorte de 103 patients porteurs de fractures tassements vertébrales dont 22 cas d'origine ostéoporotique, Slimane *et al.* avaient trouvé sur un suivi à 2 ans postopératoire, 98,3% des patients qui ne prenaient plus d'antalgiques ou qui étaient uniquement sous des paliers 1. Huit nouvelles fractures avaient été constatées chez 3 de leurs patients ostéoporotiques (2,9%). Ils n'avaient enregistré aucune fuite de ciment symptomatique et aucun événement indésirable sévère lié au dispositif ne s'est produit à 24 mois de recul [10]. Contrairement à cette étude, nous n'avons pas enregistré de fracture des vertèbres adjacentes, mais 3 cas de fuite de ciment ont été trouvés. La fuite du ciment pourrait être dû soit à une pression de poussée importante lors de l'injection du ciment qui se fait le plus souvent manuellement; ou à l'injection de celui-ci avant le début de sa polymérisation ou enfin à une rupture de la corticale de la vertèbre lors de la restauration de sa hauteur. Ce procédé chirurgical entraine une disparition immédiate et permanente de la douleur pour un retour rapide aux activités quotidiennes et professionnelles [11]. Cette technique mini-invasive est moins délétère pour le patient avec une diminution importante du risque infectieux lié à la chirurgie, un temps d'intervention très réduit et un durée d'hospitalisation aussi nettement raccourcie. La tendance actuelle va vers une chirurgie sous anesthésie locale avec une sédation légère et en ambulatoire.

## Conclusion

La kyphoplastie percutanée avec les implants de 3^e^ génération comme le SpineJack® a simplifié le traitement chirurgical des fractures ostéoporotiques vertébrales. Le degré d'ouverture suffisant du SpineJack® offre une réduction pérenne consolidée par le ciment. Des études avec une cohorte beaucoup importante seront nécessaires avec un recul suffisant pour apprécier le comportement de cet implant ainsi que l'état de la statique rachidienne après une kyphoplastie avec SpineJack®.

### Etat des connaissances actuelles sur le sujet

- Les fractures ostéoporotiques rachidiennes sont très invalidantes;
- Les rhumatologues et les internistes sont pour la plupart réticents à un traitement chirurgical. Pour les patients opérés, la vertébroplastie était la technique la plus proposée; celle-ci consiste à consolider la fracture à l'aide du ciment acrylique mais sans redonner la hauteur vertébrale;
- Avec la kyphoplastie au ballonnet, une perte de hauteur secondaire de la hauteur vertèbre survient après le dégonflage des ballonnets et avant l'injection du ciment.

### Contribution de notre étude à la connaissance

- La grande ouverture des ailettes de l'extenseur en titane permet d'obtenir une réduction optimale de la fracture vertébrale;
- Notre étude renforce des données proactives en faveur d'indication chirurgicale plutôt qu'un traitement conservateur;
- Cette chirurgie pour être efficace devra adopter la technique de la kyphoplastie avec des extenseurs vertébraux à large ouverture.

## Conflits d’intérêts

Les auteurs ne déclarent aucun conflit d'intérêts.
